# Sleep and Cardiometabolic Health: Integration Into Internal Medicine Practice

**DOI:** 10.7759/cureus.111756

**Published:** 2026-06-29

**Authors:** Sofia Almeida, Pedro M Neves, Vital Da Silva Domingues

**Affiliations:** 1 Internal Medicine, Unidade Local de Saúde de Santo António, Porto, PRT

**Keywords:** cardiometabolic risk, hypertension, internal medicine, obstructive sleep apnea, sleep, type 2 diabetes

## Abstract

Sleep is a critical and often underestimated determinant of cardiometabolic health, influencing neuroendocrine, inflammatory, and metabolic pathways. Sleep disorders are associated with an increased risk of cardiovascular and metabolic diseases, including hypertension, type 2 diabetes, and obesity. This article aims to review the main pathophysiological mechanisms linking sleep to cardiometabolic disease, summarize the most recent clinical evidence, and propose intervention strategies applicable to Internal Medicine practice. We conducted a narrative review of literature published between 2015 and 2025, including observational studies, clinical trials, meta-analyses, and international guidelines, with a focus on content relevant to Internal Medicine settings. Recent studies report that high-risk sleep patterns are associated with a 30-60% increased risk of cardiovascular events. Interventions like cognitive behavioral therapy for insomnia (CBT-I), continuous positive airway pressure (CPAP), and sleep hygiene education have demonstrated clinical benefit. Systematic evaluation and targeted intervention on sleep should be incorporated into the internist's routine care. Recognizing sleep as a modifiable risk factor is essential for evidence-based, patient-centered care.

## Introduction and background

Cardiovascular and metabolic diseases remain among the leading causes of morbidity and mortality worldwide. The World Health Organization (WHO) estimates that cardiovascular diseases account for around 32% of all global deaths in 2022 [1]. In Portugal, the scenario is similar, with recent data from Statistics Portugal (Instituto Nacional de Estatística (INE)) indicating that, in 2022, diseases of the circulatory system accounted for approximately 26.6% of deaths in the country [2]. This scenario reflects a high prevalence of modifiable risk factors, such as arterial hypertension, dyslipidemia, obesity, and type 2 diabetes mellitus, within the Portuguese adult population [3].

Despite advances in the management of these classical factors, sleep has emerged over the past two decades as a critical and frequently underestimated determinant of cardiometabolic health [4]. Sleep disturbances, including chronic insomnia, obstructive sleep apnea (OSA), and voluntary sleep restriction, have demonstrated adverse effects on neuroendocrine function, glycemic homeostasis, autonomic regulation, and inflammatory response [5-7]. These alterations appear to contribute to the development and worsening of conditions such as hypertension, type 2 diabetes, dyslipidemia, and coronary artery disease [5,6,8,9].

Despite the robustness of these data, sleep assessment remains scarce in routine clinical practice [10], particularly in Internal Medicine, where multimorbidity and longitudinal follow-up would favor its integration. Sleep continues to be undervalued during medical history-taking and is rarely included as a therapeutic target in cardiometabolic risk management plans [11].

This review article aims to synthesize the main pathophysiological mechanisms linking sleep to cardiometabolic disease, analyze the most up-to-date clinical evidence, and propose practical strategies for assessment and intervention applicable to Internal Medicine.

## Review

Methods of the narrative review

We conducted a narrative review of literature using PubMed, Scopus, and Web of Science databases, including observational studies, clinical trials, meta-analyses, and international guidelines involving adult populations, published in English or Portuguese, with a focus on content relevant to Internal Medicine settings published between January 2015 and December 2025. Recommendations from relevant scientific societies, such as the American Heart Association or the American Academy of Sleep Medicine, were also reviewed to contextualize the available evidence.

Search terms included combinations of the descriptors “sleep disturbances," "insomnia," “obstructive sleep apnea," "inflammation," “cardiovascular diseases," and “metabolic diseases."

The limitations inherent to narrative reviews are acknowledged, including potential selection bias and the absence of quantitative synthesis. However, this approach allows the integration of heterogeneous evidence into a clinically meaningful framework applicable to Internal Medicine practice.

Sleep as a determinant of cardiometabolic health

Sleep plays a fundamental role in regulating multiple physiological systems that directly influence cardiovascular and metabolic health [4,5]. Multiple mechanisms are involved, namely, the autonomic nervous system regulation, the blood pressure regulation, the metabolic and glucose regulation, the inflammatory pathways, and the neuroendocrine effects [5,11-13].

According to international guidelines and recent systematic reviews, adequate duration (seven to nine hours per night in adults), continuity, and sleep quality are essential for autonomic homeostasis, hormonal balance, immune function, and glucose and lipid metabolism [14,15].

Various sleep disorders, including chronic insomnia, OSA, social jet lag, and voluntary sleep deprivation, contribute to neurohormonal, inflammatory, and metabolic dysfunction. These disturbances affect blood pressure variability, promote insulin resistance and dyslipidemia, and increase visceral adiposity. The pathophysiological mechanisms for these disturbances can be summarized in Table 1.

**Table 1:** Sleep disorders and associated pathophysiological mechanisms

Sleep disorder	Pathophysiological mechanisms	Blood pressure (BP) variability/hypertension	Insulin resistance	Dyslipidemia	Visceral adiposity	Supporting references
Obstructive sleep apnea (OSA)	Intermittent hypoxia → sympathetic activation; oxidative stress; endothelial dysfunction; systemic inflammation; sleep fragmentation	Increased sympathetic drive and impaired nocturnal BP dipping	Hypoxia- and inflammation-mediated impairment of insulin signaling	Dysregulated lipid metabolism secondary to inflammation and hypoxia	Promotes visceral fat accumulation via metabolic dysregulation and leptin-related pathways	[4,5,6,7,11,12]
Social jet lag/circadian misalignment	Circadian rhythm disruption; central-peripheral clock desynchronization; hormonal misalignment (cortisol, melatonin); irregular sleep–wake timing	Altered circadian BP rhythm and reduced nocturnal dipping	Impaired glucose tolerance due to misaligned feeding–sleep cycles and circadian disruption	Hepatic lipid metabolism dysregulation and increased cardiometabolic risk	Energy imbalance and circadian-driven adiposity accumulation	[4,5,11,14,15]
Chronic insomnia	Hyperarousal state; hypothalamic-pituitary-adrenal axis activation; sympathetic overactivity; increased inflammatory signaling; fragmented sleep	Elevated resting BP and impaired nocturnal BP dipping	Cortisol-mediated insulin resistance and inflammatory impairment of glucose metabolism	A pro-inflammatory state contributing to an adverse lipid profile	Indirect effect via chronic stress and cortisol-driven fat redistribution	[7,10-13]
Voluntary sleep deprivation/short sleep duration	Sleep curtailment → neuroendocrine dysregulation (↓leptin, ↑ghrelin); increased appetite; sympathetic activation; systemic inflammation	Increased BP and reduced dipping due to an autonomic imbalance	Reduced insulin sensitivity and impaired glucose tolerance (experimental evidence)	Metabolic dysregulation leading to a pro-atherogenic lipid profile	Increased caloric intake and hormonal imbalance → visceral fat gain	[4,8,9,11,13-15]

Furthermore, sleep fragmentation and non-restorative sleep have been associated with greater baseline sympathetic activation, loss of circadian blood pressure variability, and increased resting heart rate, factors that elevate cardiovascular risk [16].

Population-based studies demonstrate that high-risk sleep patterns increase the likelihood of established cardiovascular disease by up to 40%, even after modifiable risk factors warranting systematic assessment in patients with cardiovascular risk, metabolic syndrome, or multiple comorbidities [17]. Monitoring and intervention represent concrete opportunities to improve overall chronic disease control [18].

Pathophysiological mechanisms

The link between sleep disturbances and cardiometabolic risk results from the coordinated activation of multiple pathophysiological mechanisms [12,13]. These alterations can occur both in situations of acute sleep deprivation and in chronic disorders such as insomnia or OSA (Table 2) [12,13].

**Table 2:** Relationship between sleep disturbances, cardiometabolic risk, and pathophysiological mechanisms

Sleep disturbance pattern	Key pathophysiological mechanisms	Blood pressure (BP) variability	Insulin resistance	Dyslipidemia	Visceral adiposity	Supporting references
Acute sleep disturbance/short-term sleep restriction	Increased sympathetic nervous system activity; acute inflammatory response; transient endothelial dysfunction; early neuroendocrine activation	Acute elevation in blood pressure and reduced nocturnal dipping patterns	Short-term reduction in insulin sensitivity and impaired glucose metabolism (experimental evidence)	Early metabolic dysregulation affecting lipid handling	No immediate structural adiposity change, but promotes positive energy balance via stress-related mechanisms	[12,13]
Chronic sleep disturbance (insomnia, chronic short sleep, fragmented sleep)	Sustained sympathetic activation; chronic low-grade inflammation; persistent hypothalamic-pituitary-adrenal axis dysregulation; endothelial dysfunction; cumulative sleep debt	Sustained hypertension risk and increased long-term BP variability	Chronic insulin resistance due to inflammatory and neuroendocrine dysregulation	Atherogenic lipid profile associated with chronic inflammatory state	Progressive increase in visceral adiposity via long-term metabolic and hormonal dysregulation	[12,13]

Firstly, sleep fragmentation and reduced deep sleep (stage N3) induce activation of the sympathetic nervous system, with sustained increases in norepinephrine, cortisol, and other catecholamines [16]. This state is associated with arterial hypertension, baroreceptor dysfunction, and elevated resting heart rate [7].

In parallel, a state of low-grade systemic inflammation is observed, characterized by elevated markers such as C-reactive protein (CRP), interleukin-6 (IL-6), and tumor necrosis factor alpha (TNF-α) [7]. These cytokines promote endothelial dysfunction, insulin resistance, and atherosclerosis progression [8,19].

From a metabolic perspective, short-term sleep restriction reduces insulin sensitivity, impairs glucose tolerance, and alters the hormonal profile regulating appetite, with decreased leptin and increased ghrelin levels, thereby facilitating hyperphagia and weight gain [8,19].

In OSA, these effects are exacerbated by intermittent nocturnal hypoxia and repeated micro-arousals, which induce oxidative stress, arterial stiffness, and left ventricular hypertrophy, significantly increasing the risk of major cardiovascular events [20].

Acting synergistically and cumulatively, these mechanisms promote a high-risk cardiovascular and metabolic phenotype that should be identified early and addressed through sleep-focused interventions [4].

Clinical and epidemiological evidence

The association between sleep disturbances and cardiometabolic risk has been demonstrated by observational studies, population cohorts, and meta-analyses [17,21]. A recent study revealed a U-shaped relationship between sleep duration and cardiovascular mortality, with a higher risk associated with durations of <6 hours or >9 hours per night [22].

Data from the National Health and Nutrition Examination Survey (NHANES) 2015-2018 show that individuals with high-risk sleep patterns, including frequent fragmentation, insomnia, and daytime sleepiness, have a higher risk of established cardiovascular disease, even after adjustment for age, sex, and traditional risk factors [23,24].

Regarding OSA, the evidence is even more robust. A 2024 study, which included 1,007 Asian adults undergoing non-emergent coronary artery bypass grafting (CABG), who underwent preoperative overnight sleep studies to assess sleep apnea, showed that patients with OSA have a 30% increased rate for major adverse cardiac and cerebrovascular events and a 60% increased hazard rate for heart failure hospitalization [20].

Consistent continuous positive airway pressure (CPAP) use significantly reduced these events, particularly when used for ≥4 hours per night [25,26].

In Portugal, the Portuguese Hypertension and Salt (PHYSA) Study revealed that more than 20% of adults report excessive daytime sleepiness and 43% exhibit patterns compatible with poor sleep quality, a clear reflection of the prevalence of undiagnosed sleep disorders in the general population [27].

These data support the integration of sleep assessment into cardiovascular and metabolic risk stratification algorithms, particularly in settings such as Internal Medicine, where multimorbidity is the norm [4]. However, the interpretation of these associations should take into account the potential influence of important confounding variables, including obesity, mental health disorders such as anxiety and depression, and socioeconomic determinants of health, all of which may independently affect both sleep quality and cardiometabolic risk. Adjusting for these factors is therefore essential to better clarify the independent contribution of sleep disturbances to adverse clinical outcomes and to improve the accuracy and applicability of risk prediction models.

Sleep-based therapeutic interventions

The therapeutic management of sleep disorders represents a concrete opportunity to intervene on a frequently neglected risk factor with direct impact on cardiometabolic health [18,28,29]. Its integration into clinical practice should be personalized, multidisciplinary, and tailored to the type of disturbance identified [28,29].

In chronic insomnia, cognitive behavioral therapy for insomnia (CBT-I) is the first-line treatment recommended by major scientific societies [28]. In a 2021 Chinese study, including 1033 individuals with type 2 diabetes and poor sleep quality, CBT-I has demonstrated effectiveness in improving sleep quality and continuity, reducing blood pressure, and enhancing glycemic control in patients with type 2 diabetes [30]. It is superior to pharmacotherapy in the medium to long term, with a lower risk of adverse effects [2].

For patients with OSA, CPAP remains the primary intervention [25,26]. Adequate adherence to CPAP (≥4 hours per night) is associated with significant improvements in blood pressure control, daytime sleepiness, and reduction in cardiovascular events [25,26]. However, adherence is variable, and follow-up should include motivational support strategies and education [29].

Sleep hygiene education should be standard practice across all clinical settings [29]. This includes recommendations on regular sleep schedules, limiting exposure to screens and stimulants, and promoting a sleep-conducive environment [29]. This intervention is particularly effective in voluntary sleep deprivation or in patients with social jet lag [18,31].

In shift-work contexts, counseling should include circadian adaptation techniques such as light modulation protocols, as established at the Working Time Society consensus; progressive schedule adjustments; and, when necessary, referral for occupational support [31,32].

Different sleep disorders involve distinct pathophysiological mechanisms, with variable impacts on cardiometabolic risk [4]. Table 3 summarizes the main conditions, their associated mechanisms, relevant diseases, and recommended interventions.

**Table 3:** Sleep disturbances and associated risk (key mechanisms)

Sleep disorder	Main mechanism	Associated conditions	Recommended interventions	References
Chronic insomnia	Sympathetic activation, sleep fragmentation	Hypertension, type 2 diabetes mellitus	Cognitive behavioral therapy for insomnia (CBT-I), sleep hygiene	[28,33]
Obstructive sleep apnea (OSA)	Intermittent hypoxia, oxidative stress	Coronary artery disease, stroke, heart failure	Continuous positive airway pressure (CPAP), weight loss, specialist referral	[25,26,34,35]
Social jet lag/shift work	Circadian disruption, insomnia	Dyslipidemia, insulin resistance	Schedule reorganization, occupational support	[31]

Ultimately, integrating these strategies into the therapeutic plan of the chronically ill patient, under the coordination of the internist, contributes to a more comprehensive, preventive, and person-centered approach [4].

The internist’s role in an integrated approach

Given their comprehensive perspective and central role in managing multimorbidity, internists are well-positioned to detect and intervene early in sleep disturbances with cardiometabolic impact [4]. Their approach should include systematic assessment, initial counseling, and referral whenever necessary [36].

A targeted clinical history should include simple, specific questions: “On average, how many hours do you sleep?" "Do you feel rested upon waking?" "Do you have difficulty maintaining sleep?” These questions allow for an initial screening of relevant alterations [36].

To support decision-making, validated and easily applicable tools exist for Internal Medicine: the Epworth Sleepiness Scale, which assesses daytime sleepiness; the Pittsburgh Sleep Quality Index (PSQI), which evaluates overall sleep quality; STOP-Bang, which is useful for screening OSA; and the Mini Sleep Questionnaire (MSQ), useful for evaluating sleep quality [36-39].

Internists can initiate basic interventions (e.g., sleep hygiene, routine education), monitor adherence to therapies such as CPAP, and ensure coordination with other specialties (pulmonology, psychology, sleep medicine) [29].

Finally, internists also play an educational role, promoting sleep health literacy, dispelling common misconceptions, and encouraging sustained behavioral change [18,29]. Integrating sleep into clinical evaluation contributes to a more holistic, person-centered, and evidence-based practice, reinforcing the fundamental values of Internal Medicine [4].

## Conclusions

Recent literature suggests that short duration, poor quality, and fragmented sleep are risk factos for cardiovascular events and mortality, even in patients without a prior diagnosis of sleep disorders. Interventions such as CBT-I, appropriate CPAP use, and structured sleep hygiene education have shown consistent clinical benefits in the prevention and management of chronic disease.

Within Internal Medicine, systematic sleep assessment should be integrated into routine practice as part of a person-centred approach and cardiovascular risk stratification. However, its implementation faces significant practical barriers, including limited consultation time, competing clinical priorities, and variability in access to validated screening tools and downstream diagnostic resources (such as polysomnography). Internists should adopt a proactive role in identifying, intervening early, and guiding the therapeutic management of sleep disorders, reinforcing their importance as a transversal pillar of health. Promoting sleep health ultimately means promoting a more preventive, effective, and truly patient-centred form of medicine.
